# Elastofibroma dorsi: A soft tissue masquerade

**DOI:** 10.4103/0973-6042.79797

**Published:** 2010

**Authors:** Pauline H. Go, Michael C. Meadows, Essel Marie B. deLeon, Ronald S. Chamberlain

**Affiliations:** 1 Saint George’s University, School of Medicine, Grenada, West Indies; 2 Department of Surgery, Saint Barnabas Medical Center, Livingston, New Jersey, USA; 3 Department of Pathology, Saint Barnabas Medical Center, Livingston, New Jersey, USA; 4 Department of Surgery, University of Medicine and Dentistry of New Jersey, Newark, New Jersey, USA

**Keywords:** Elastofibroma dorsi, fibroma, hemangioma, lipoma, sarcoma, soft tissue tumor, subscapular mass

## Abstract

Elastofibroma dorsi (ED) is a soft tissue tumor found in the subscapular region. The pathogenesis of ED is unclear, but may involve a regenerative or reactive hyperproliferation due to mechanical microtrauma. Magnetic resonance imaging (MRI) is preferred to diagnose ED and complete excision is curative. When bilateral, subscapular masses are identified in the elderly patient and MRI characteristics are typical, biopsy and excision can be avoided. Symptomatic EDs should be excised, and recurrence is rare. Three hundred and thirty cases of ED have been reported since 1980. Fourteen case series and 43 isolated case reports involved 263 women and 67 men (F:M ratio = 3.9:1), with a mean age of 62 years (range 6–94 years). Bilateral ED was present in 164 patients and unilateral ED in 157. The reported prevalence in the elderly population ranges from a minimum of 2% to a maximum of 24%.

## INTRODUCTION

Elastofibroma dorsi (ED) is a benign, slow-growing fibroelastic tumor that was first described by Jarvi and Saxen in 1961.[1] The typical presentation of ED involves a subscapular mass associated with a long history of swelling, discomfort, snapping of the scapula and, in some cases, pain. In 99% of cases, EDs are localized to the infrascapular region between the thoracic wall, serratus anterior, lattisimus dorsi muscle, and are often attached to the periosteum of the thoracic wall.[1–3] EDs typically appear as a solitary, poorly circumscribed, heterogeneous, soft tissue mass on magnetic resonance imaging (MRI). The differential diagnosis includes lipomas, liposarcomas, fibromas, hemangiomas, and hematomas.[4] While definitive biopsy is necessary to confirm the diagnosis in cases of unilateral EDs, bilateral subscapular lesions exhibiting classic MR findings in older patients are often sufficient for a presumptive diagnosis.[3,4]

Three hundred and thirty cases of symptomatic ED have been reported in the last 30 years. A comprehensive review of all isolated reports and case series, published since 1980, affirms that EDs are diagnosed almost exclusively in persons over the age of 40, with sporadic reports of ED arising in children and adolescents as young as 6 years.[4–6] The prevalence of these tumors is reported to be as low as 2%[5] and as high as 24%[2] of the elderly population (>55 years old). Data from 14 case series published between 1980 and 2009 have been compiled in Table 1. These studies involved 263 women and 67 men (F:M ratio=3.9:1), with an overall mean age of 62 years at diagnosis (range 6–94 years). Bilateral ED was found in 164 patients and unilateral ED in 157 (U:B ratio=1:1) patients. All the patients who underwent excision recovered fully with no loss in range of motion. Six patients experienced recurrence 6 months to 17 years after surgery, but no case of malignant transformation has been reported to date.

**Table 1:** Published elastofibroma dorsi case series from 1980 to 2009

Study	N	Gender (F:M)	Mean age at diagnosis	Unilateral: bilateral
Burton *et al*., 2009[13]	6	2:1	65	5:1
Cinar *et al*., 2009[14]	13	11:2	54	11:2
Kastner *et al*., 2009[15]	11	9:2	62	10:1
Chandrasekar *et al*., 2008[11]	15	1:4	68	13:2
Muratori *et al*., 2008[7]	8	7:1	61	7:1
Daigeler *et al*., 2007[12]	7	5:2	64	6:1
Mortman *et al*., 2007[16]	6	1:1	NR	6:0
Muramatsu *et al*., 2007[17]	8	5:3	67	3:1
Vastamaki *et al*., 2001[18]	5	3:2	62	4:1
Majó *et al*., 2001[19]	10	3:2	57	3:2
Briccoli *et al*., 2000[20]	9	5:4	60	2:1
Naylor *et al*., 1996[3]	12	5:1	65	1:3
Marin *et al*., 1989[10]	7	4:3	49	5:2
Nagamine *et al*., 1982[6]	170	13:1^a^	70	1:2^b^

*N*: sample size of study; NR: not reported;

a Actual distribution of gender: 158 females, 12 males;

b Actual distribution of sidedness: 57 unilateral, 112 bilateral

We report our experience treating an illustrative case of ED and provide a discussion of the significance of physical exam and imaging in the diagnosis of these tumors in order to avoid surgery.

## CASE REPORT

A 55-year-old male machinist with no significant medical or surgical history presented with a right subscapular mass causing mild discomfort. He denied debilitating pain, recent trauma, or weight loss. Physical examination revealed a small, 4 cm mass, inferior to the right scapular spine. Laboratory findings were unremarkable. Computed tomography (CT) imaging of the chest with contrast revealed an 8×2.1 cm soft tissue mass, similar in density to muscle, that was located superficial to the ribs and deep to the serratus anterior and lower subscapularis muscles. The lesion was slightly inhomogenous, non-enhancing and contained strands of fatty tissue. A much smaller (1×1.5 cm) contralateral lesion was also noted at that time. Histopathology of samples obtained by core biopsy and a subsequent incisional biopsy revealed normal muscle and adipose tissue and no further intervention was performed.

**Figure 1:**
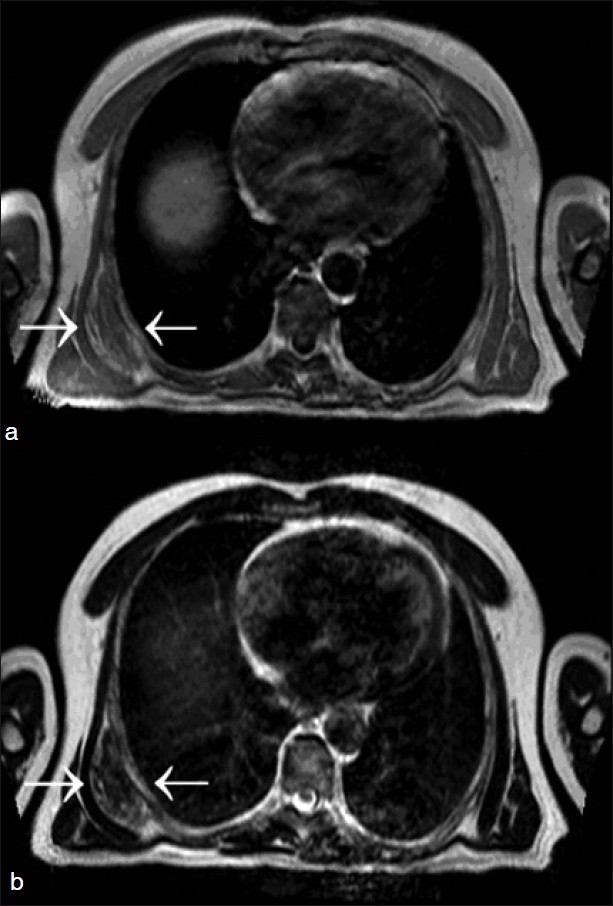
MRI of a subscapular elastofibroma from our patient is shown. (a) T1- and (b) T2-weighted images showing the typical well-defined, inhomogenous mass (arrows), with signal intensity similar to that of surrounding skeletal muscle and interlaced with areas of adipose tissue

The patient returned 2 years later with increasing discomfort and limited range of motion on the right side. MRI revealed a mass below the body of the scapula, which was isointense to muscle and contained interspersed signals similar to fat, with moderate diffuse enhancement seen on post-gadolinium imaging [Figure 1]. ED was diagnosed at this time and complete excision of the symptomatic right subscapular mass was performed. Six weeks following surgery, the patient fully recovered.

**Figure 2:**
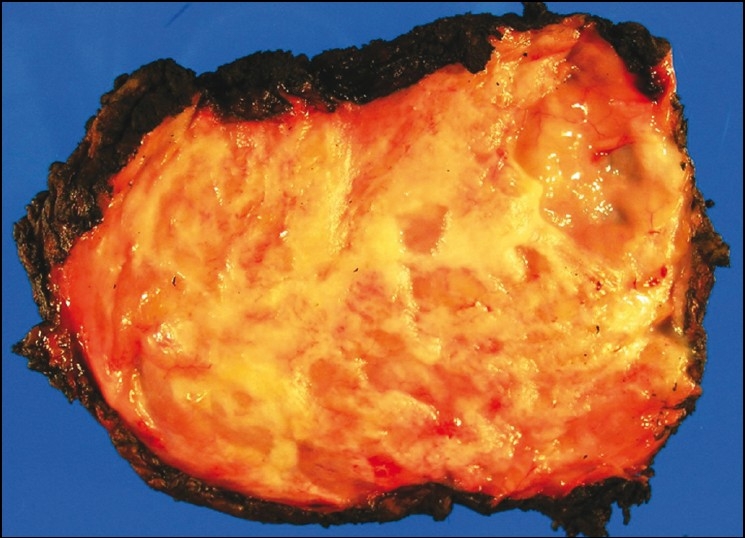
Macroscopic appearance of a subscapular elastofibroma removed from our patient. The tumor was a poorly circumscribed mass showing white soft to rubbery areas admixed with adipose tissue

**Figure 3:**
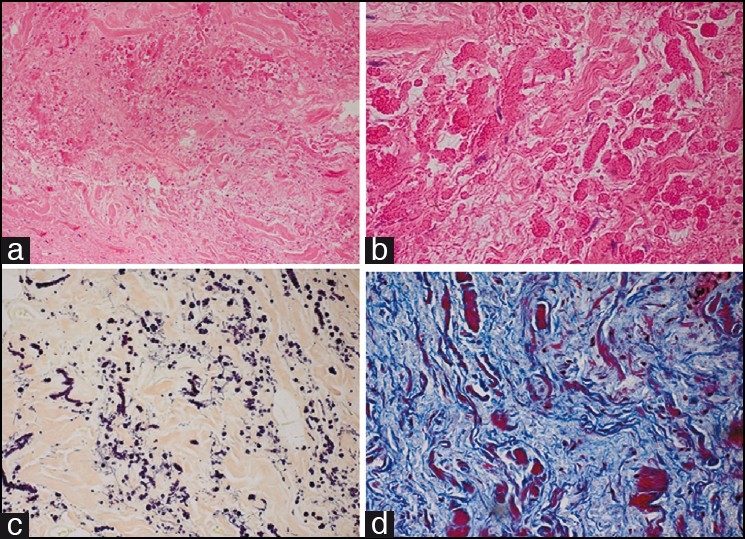
Histopathologic and immunohistochemical findings of an elastofibroma removed from our patient: (a) Haphazardly arranged fascicles of elastic fibers in a collagenous matrix (hematoxylin–eosin, ×20 magnification); (b) brightly eosinophilic elastic fibers with serrated edge globules (hematoxylin–eosin, ×40); (c) elastin stain highlights the disorganized elastic fibers, ×20; (d) trichrome stain outlines the collagenous matrix, ×40

The resected specimen consisted of a single, irregular, pink-red fragment of soft tissue that measured 10×6×2.5 cm and weighed 81 g. The cut surface varied from pink to white in color and was soft to rubbery in consistency with focal cystic degeneration [Figure 2]. Histopathology demonstrated a poorly circumscribed proliferation of randomly arranged fascicles of fibrous tissue interspersed with islands of adipose tissue on hemotoxylin and eosin (H and E) staining [Figure 3a and b] and disorganized elastic and collagen fibers on elastin and trichrome stains [Figure 3c and d]. There was no necrosis, atypia, or increased mitotic activity identified.

## DISCUSSION

First described by Jarvi and Saxen in 1961, ED is a benign soft tissue tumor located in the subscapular and infrascapular region between the thoracic wall, serratus anterior, and lattisimus dorsi muscle.[1] Rare occurrences in the ischial tuberosities, hand, foot, orbit, mediastinum, intraspinal spaces, greater omentum, and stomach have also been reported.[6] Swelling, discomfort, snapping of the scapula, and occasionally pain are the most common presenting symptoms. However, it is not uncommon for these tumors to be completely asymptomatic and to be found incidentally during physical examination or during an investigation of a symptomatic mass on the contralateral side.

The etiology of ED is not fully understood, but most believe that ED results from degeneration of collagen due to repeated mechanical friction between the chest wall and scapular tip, while others have suggested that it may be the cause of a reactive hyperproliferation of fibroblastic tissue rather than a degenerative process.[2,7] Geibel *et al*. speculated that ED may be the result of a physiologic aging process, rather than abnormal elastogenesis or degeneration, based on the discovery of “pre-elastofibroma changes” in their autopsy series. These changes are defined by a weakly elastinophilic material that does not exhibit definite elastic tissue formation.[8] Additional confirmatory literature on this theory is scant.[9] Nagamine *et al*. have reported a familial predisposition for ED, noting that 32% of their cases occurred within a single family.[6] To date, it is universally accepted that EDs are more common in persons who perform repetitive manual labor involving the shoulder girdle though the mechanism is unclear.[2,6,10]

Historically, ED has been regarded as rare tumors occurring predominantly in the elderly. Brandser *et al*. performed a CT study of 258 asymptomatic elderly patients and identified ED in 2% of the patients.[5] With the exception of a single large series by Nagamine *et al*. (*n* = 170),[6] the literature on ED consists mostly of isolated case reports and small series, furthering the misconception that ED is a rare condition. However, when accounting for incidental findings of ED and those discovered on autopsy, the prevalence may be much higher. Jarvi *et al*. found ED in 24% of females and 11% of males in people over 55 years of age in a large autopsy series involving 235 cases.[2] In this study, all masses were less than 3 cm in size and too small to be seen grossly on physical examination or to produce symptoms that would prompt the patient to seek medical attention. Giebel *et al*. reported that ED occurred in 13% and pre-elastofibroma changes in 81% among autopsies performed on 100 patients.[8] Moreover, Naylor *et al*. reported incidental findings of ED in 12 out of 15 patients, nine of whom were diagnosed by CT/MR imaging for symptoms of ED on the contralateral side, and three were diagnosed during surgery for an unrelated thoracic disease.[3] A small ED lesion was found incidentally in our own patient on the contralateral side during imaging workup for the symptomatic right subscapular mass. It is reasonable to conclude that the majority of EDs are small and clinically silent, creating the false perception that the prevalence is small.

An understanding of the classic imaging features of ED is vital to make a presumptive diagnosis and eliminate the need for biopsy or unnecessary surgical resection. MR is the imaging modality of choice in diagnosing ED and typically demonstrates a well-defined, moderately inhomogenous mass with no associated soft tissue edema. On T1-weighted MRI, EDs are isointense with skeletal muscle, which explains how these tumors are often diagnostically overlooked. T1- and T2-weighted images both show interspersed linear and curvilinear, hyperintense areas representing fat.[3,4,11] Fat saturation gradient technique applied post-gadolinium administration may show subtle areas of heterogenous enhancement within the mass, making it difficult to exclude a soft tissue sarcoma without biopsy.[3,4,7,11] Imaging with CT often reveals homogenous masses with attenuation similar to that of surrounding skeletal muscle and a fat plane that is indistinct from the mass and adjacent skeletal muscle.[5] As demonstrated in our illustrative case, CT lacks the necessary contrast resolution to differentiate streaks of abnormal elastic tissue from normal tissue and will only demonstrate an ED if the mass is sufficiently large.[5] A presumptive diagnosis can be made in an elderly patient who presents with bilateral soft tissue masses located in the subscapular region, which exhibit the classic radiographic appearance of an ED as described above.[3,4] In such cases, a biopsy is not obligatory. In less clear-cut cases, a core or open biopsy is essential to make a definitive diagnosis.

Macroscopically, elastofibromas appear as an irregular, poorly defined, unencapsulated, fibroelastic mass with firm, rubbery consistency. Cut surface reveals strands of white and yellow tissue representing adipose tissue intermingled with fibroelastic tissue in a “checkerboard” pattern.[3] Histologically, fibrous, collagenous strands with eosinophilic, plump, elongated and round-shaped collections of elastic fibers are seen. These fibers may be difficult to visualize on H and E, especially during frozen section diagnosis. An elastin stain (Elastica-van-Gieson) is necessary to highlight these fibers and reveals deeply staining branched and unbranched fibers with central dense core and serrated margins. The lesions are predominantly hypocellular, with benign fibrocytic and fibroblastic cells that lack atypia and mitotic figures, and contain entrapped islands of adipose tissue.[3,4,6,11,12] Fine needle aspiration is not a recommended diagnostic technique due to this hypocellular nature of ED.[12]

**Figure 4:**
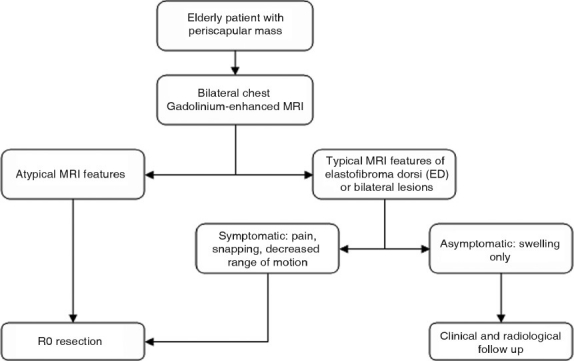
Diagnostic and therapeutic algorithm for treating patients with periscapular mass[17] (MRI = Magnetic resonance imaging)

Excision may be offered to symptomatic patients, with curative marginal resection proving to be sufficient and is preferred over wide or radical resection.[12] Because of their benign and indolent nature, there is no need to treat asymptomatic tumors if the diagnosis of ED can be confirmed. Muratori *et al*. suggested an algorithm for the diagnosis and treatment of a subscapular elastofibromal mass,[7] which correlates well with our own approach on second evaluation [Figure 4]. In the case of a single asymptomatic mass that demonstrates typical MR features, as previously discussed, or bilateral masses in an elderly patient, clinical follow-up is sufficient. On the other hand, in the case of a symptomatic mass with typical MR features or an asymptomatic mass with atypical MR features, marginal resection is indicated.

All patients who underwent a marginal R0 resection (negative macroscopic and microscopic margins) were free of disease at follow-up and experienced no loss in range of motion.[3,6,7,10,20] The few reported recurrences in the literature were presumed to be the result of incomplete excision.[4,6] No cases of malignant transformation have been reported.[12]

In conclusion, our case report initially confirmed how CT imaging and general lack of awareness of this mass can lead to a missed diagnosis despite biopsy. Fortunately, on second evaluation by following the protocol set forth by Muratori *et al*.[7] in which MR, the preferred imaging modality to locate and characterize this lesion, was utilized, we were able to successfully diagnose this patient and definitively treat via an R0 resection. Furthermore, given the findings on MRI of an inhomogenous lesion that is similar in appearance to skeletal muscle with interspersed areas of adipose tissue, in an elderly patient or patients with bilateral lesions, a presumptive diagnosis can be made without the need for biopsy. In all other cases, an open biopsy is justified to rule out malignancy and reassure an asymptomatic patient that no further surgical intervention is needed. R0 resection is sufficient to cure symptomatic and asymptomatic patients.
